# Pharmacodynamic rationale for the choice of antiseizure medications in the paediatric population

**DOI:** 10.1016/j.neurot.2024.e00344

**Published:** 2024-03-22

**Authors:** Gianluca D'Onofrio, Roberta Roberti, Antonella Riva, Emilio Russo, Alberto Verrotti, Pasquale Striano, Vincenzo Belcastro

**Affiliations:** a Department of Neurosciences Rehabilitation, Ophthalmology, Genetics, Maternal and Child Health (DiNOGMI), University of Genoa, Via Gerolamo Gaslini 5, 16147 Genoa, Italy; b Science of Health Department, Magna Græcia University, Catanzaro, Italy; c Department of Pediatrics, University of Perugia, Perugia, Italy; d Pediatric Neurology and Muscular Diseases Unit, IRCCS Istituto “Giannina Gaslini”, Via Gerolamo Gaslini 5, 16147 Genoa, Italy; e Neurology Unit, Maggiore Hospital, Lodi, Italy

**Keywords:** Epilepsy, Interactions, Anti-epileptic drugs, Mechanism of action, Polytherapy, Treatment

## Abstract

In the landscape of paediatric epilepsy treatment, over 20 anti-seizure medications (ASMs) have gained approval from Drug Regulatory Agencies, each delineating clear indications. However, the complexity of managing drug-resistant epilepsy often necessitates the concurrent use of multiple medications. This therapeutic challenge highlights a notable gap: the absence of standardized guidelines, compelling clinicians to rely on empirical clinical experience when selecting combination therapies. This comprehensive review aims to explore current evidence elucidating the preferential utilization of specific ASMs or their combinations, with a primary emphasis on pharmacodynamic considerations. The fundamental objective underlying rational polytherapy is the strategic combination of medications, harnessing diverse mechanisms of action to optimize efficacy while mitigating shared side effects. Moreover, the intricate interplay between epilepsy and comorbidities partly may influence the treatment selection process. Despite advancements, unresolved queries persist, notably concerning the mechanisms underpinning drug resistance and the paradoxical exacerbation of seizures. By synthesizing existing evidence and addressing pertinent unresolved issues, this review aims to contribute to the evolving landscape of paediatric epilepsy treatment strategies, paving the way for more informed and efficacious therapeutic interventions.

## Introduction

Epilepsy is a chronic neurological disorder characterised by a predisposition to generate epileptic seizures. It affects more than 10 million children worldwide [1]. According to the definition of the International League Against Epilepsy (ILAE), epilepsy is diagnosed when two unprovoked seizures separated by more than 24 ​h from each other occurred or when, following a first unprovoked seizure, the risk of recurrence at 10 years is at least 60% [2]. Likewise, epilepsy is diagnosed if clinical and electroencephalographic features fulfil the criteria for an epileptic syndrome [2].

Epilepsy treatment is mainly symptomatic and relies on the chronic administration of antiseizure medications (ASMs), aimed at controlling seizures [3]. In clinical practice, the introduction of an ASM is appropriate when the seizure recurrence risk is more than 50% [4]. The term ASM is currently preferred to the previous definitions of antiepileptic or anticonvulsant drugs. ASMs have not been shown to impact epileptogenesis, and not all epileptic seizures present with a convulsive activity [5].

Firstly, the choice of an ASM depends on the semiology of the epileptic seizure and/or the type of epileptic syndrome being treated, as well as on the possible occurrence of patient comorbidities and the drug's safety profile [6,7]. The ILAE's 2017 operational classification of the epilepsies placed seizures semiology diagnosis at the top level, followed by the correct type of epilepsy (focal, generalized, or with combined mechanism) and epilepsy syndrome when possible [8].

Successful seizure control is reached with ASM monotherapy in about 50 % of patients [9]. However, in the remainder of cases, polytherapy is necessary [10]. When two tolerated and appropriately chosen ASMs fail to achieve seizure freedom, this is termed drug-resistant epilepsy (DRE) [11]. DRE is present in about 30% of children with epilepsy, with an incidence of around 15% [12]. Despite this being widely practised, there is currently little evidence on how to optimally combine ASMs in the course of polytherapy [13].

Historically, ASMs are divided into 3 generations. The first generation includes drugs launched from the early 1900s to the 1980s, while the second and third generations embrace drugs developed and approved during the 1990s and 2000s respectively [14]. The newer ASMs differ in their mechanism of action, clinical spectrum and tolerability profile [14]. Nevertheless, all ASMs act by reducing the probability of firing and propagation of action potentials and by reducing the synchronisation of localised neurons. They thereby exert an anti-ictogenic action [15]. An exception to this paradigm is the recent everolimus approval by the Food and Drug Administration (FDA), for the treatment of focal seizures associated with tuberous sclerosis complex (TSC) [16]. This is an mTOR inhibitor, that would appear to exert an anti-epileptogenic action as well, modifying the natural history of the disease [17].

To date, although the availability of ASMs has increased exponentially in recent decades, the evidence for preferring an ASM mainly based on its mechanism of action for a given epilepsy syndrome or in DRE and its underlying polypharmacy is still limited [18,19].

Here, we aimed to critically review the most recent evidence on pharmacodynamic considerations to be taken into account in the case of paediatric ASM therapy management.

## Currently approved ASMs in paediatric age

The list of the 30 molecules currently approved by the US and European drug regulatory agencies is provided in Table 1, with the age group alongside**.** The table also summarises the available formulation(s) for each molecule, together with labelled indications regarding the type of seizure and/or epileptic syndrome for which it is approved, as well as the main mechanism of action.

**Table 1:** Currently approved anti-seizure medications in paediatric age.

Drug formulation	FDA approval for seizure type(s) and/or syndrome (age if specified)	EMA approval for seizure type(s) and/or syndrome (age if specified)	Main mechanism(s) of action
Adrenocorticotropic hormone (ACTH) Injection	Monotherapy in infantile spasms (<2 ​y)	NA	Unknown, adrenocortical secretion stimulator
Brivaracetam (BRV) Tablets Oral solution Injection	Partial onset seizures (>1 ​m)	Adjunctive therapy for partial-onset seizures with or without secondary generalisation (>2 ​y)	Binding synaptic vesicle SV2A
Cannabidiol (CBD) Oral solution	LGS, DS, TSC (>1 ​y)	Adjunctive therapy (with CLB) in LGS and DS (>2 ​y) Adjunctive therapy in TSC (>2 ​y)	GPR55 and TPRV1 channels modulator, ENT-1 inhibitor
Carbamazepine (CBZ) Tablets^(XR)^ Oral solution	Partial seizures with complex symptomatology, GTC, mixed seizure patterns (CBZ does not control absences)	Partial seizures with complex symptomatology, GTC, mixed seizure patterns (CBZ does not control absences and myoclonic seizures)	Voltage-gated sodium channels blocker (by stabilizing fast-inactivated state)
Cenobamate (CBN) Tablets	Partial-onset seizures in adult patient (>18 ​y)	Adjunctive treatment of focal-onset seizures with or without secondary generalisation in adult patients	Voltage-gated sodium channels blocker (by blocking the persistent sodium current), positive allosteric modulation of GABA_A_ receptors
Clobazam (CLB) Tablets Oral solution	Adjunctive treatment in LGS (>2 ​y)	NA	Positive allosteric modulator of GABA_A_ receptors
Clonazepam (CNZ) Tablets Oral solution Injection	LGS, atonic, myoclonic Absence who have failed succinimides (injection)	NA	Positive allosteric modulator of GABA_A_ receptors
Eslicarbazepine acetate (ESL) Tablet	Partial-onset seizures (>4 ​y)	Adjunctive therapy in partial-onset seizures with or without secondary generalisation (>6 ​y)	Voltage-gated sodium channels blocker (by stabilizing fast-inactivated state)
Ethosuximide (ETH) Capsule Oral solution	Absence epilepsy (>3 ​y)	Absence epilepsy	Low-voltage activated calcium channels (T-type) blocker
Everolimus Tablets	Adjunctive treatment of partial-onset seizures in TSC (>2 ​y)	Add-on in partial-onset seizures in TSC that have not responded to other treatments (>2 ​y)	mTOR inhibitor
Felbamate (FBM) Tablet Oral solution	Adjunctive therapy in partial and generalized seizures associated with LGS (>2 ​y)	Adjunctive therapy in partial and generalized seizures associated with LGS (>4 ​y)	NMDA receptors antagonist, positive modulating action on GABA_A_ receptors, voltage-sensitive sodium and calcium channels blocker
Fenfluramine (FFA) Oral solution	Seizures associated with DS and LGS (>2 ​y)	Adjunctive therapy in seizures associated with DS and LGS (>2 ​y)	Serotonin-releasing agent
Gabapentin (GBP) Tablet Capsule Oral solution	Adjunctive therapy in partial onset seizures, with and without secondary generalization (>3 ​y)	Adjunctive therapy in partial onset seizures, with and without secondary generalization (>6 ​y) Monotherapy in partial onset seizures, with and without secondary generalization (>12 ​y)	High voltage-activated calcium channels (P/Q type) blocker, through binding to the α2δ-1 subunit
Ganaxolone (GNX) Oral solution	Seizures associated with cyclin-dependent kinase-like 5 (CDKL5) deficiency disorder (>2 ​y)	Seizures associated with cyclin-dependent kinase-like 5 (CDKL5) deficiency disorder (2–17 ​y)	Positive allosteric modulator for both synaptic and extrasynaptic GABA_A_ receptors
Lacosamide (LCM) Tablet Oral solution Injection	Partial-onset seizures (>4 ​y) Partial-onset seizures (>17 ​y) (injection)	Monotherapy in partial-onset seizures with or without secondary generalisation (2 ​y) Adjunctive therapy in partial-onset seizures with or without secondary generalisation (2 ​y) and primary GTC seizures in patients with IGE (4 ​y)	Voltage-gated sodium channels blocker (by stabilizing slow-inactivated state)
Lamotrigine (LTG) Tablet	Adjunctive therapy in partial-onset seizures, primary GTC, generalized seizures of LGS (>2 ​y) Conversion to monotherapy in partial-onset seizures (>16 ​y)	Adjunctive treatment in partial seizures and generalized seizures, including tonic-clonic seizures and seizures associated with LGS (>2 ​y) Monotherapy of typical absence seizures (>2 ​y) Adjunctive or monotherapy treatment in partial seizures and generalized seizures, including tonic-clonic seizures and seizures associated with LGS (>13 ​y)	Voltage-gated sodium channels blocker (by stabilizing fast-inactivated state and consequently modulating presynaptic transmitter release of excitatory amino acids), calcium channels blocker (N- and P/Q-type, weakly T-type)
Levetiracetam (LEV) Tablet Oral solution Injection	Adjunctive therapy in partial-onset seizures (>1 ​m), myoclonic seizures in patients with JME (>12 ​y), primary GTC in patients with IGE (>6 ​y)	Monotherapy in partial-onset seizures with or without secondary generalisation (>16 ​y) Adjunctive therapy in partial-onset seizures (>1 ​m), myoclonic seizures in patients with JME (>12 ​y), primary GTC in patients with IGE (>12 ​y)	Binding synaptic vesicle SV2A
Oxcarbazepine (OXC) Tablet Oral solution	Partial seizures (adjunctive therapy >2 ​y, monotherapy >4 ​y)	Monotherapy or adjunctive therapy in partial seizures with or without secondarily GTC seizures (>6 ​y)	Voltage-gated sodium channels blocker (by stabilizing fast-inactivated state)
Perampanel (PER) Tablet	Partial-onset seizures with or without secondarily generalized seizures (>4 ​y); Adjunctive therapy in primary GTC (>12 ​y)	Adjunctive treatment in partial-onset seizures with or without secondarily generalized seizures (>4 ​y) Primary GTC seizures in patients with IGE (>7 ​y)	Non-competitive AMPA glutamate receptor antagonist
Phenobarbital (PHB) Tablet Oral solution Injection	Treatment of neonatal seizures in term and preterm infants (injection form)	NA	Positive allosteric modulator of GABA_A_ receptors (agonist effect at high doses)
Phenytoin (PHT) Tablet Capsule Oral solution Injection	GTC and complex partial seizures; prevention or treatment of seizures occurring during or following neurosurgery	GTC, partial seizures or a combination of these; prevention and treatment of seizures occurring during or following neurosurgery and/or severe head injury Status epilepticus of the tonic-clonic type and prevention and treatment of seizures occurring during or following neurosurgery and/or severe head injury	Voltage-gated sodium channels blocker (by stabilizing fast-inactivated state)
Pregabalin (PGB) Tablet Capsule Oral solution	Adjunctive therapy for the treatment of partial onset seizures (>4 ​y)	Add-on to existing treatment in patients who have partial seizures	High voltage-activated calcium channels (P/Q type) blocker, through binding to the α2δ-1 subunit
Primidone (PRM) Tablet	Alone or add-on in GTC, psychomotor and focal seizures	Grand mal and psychomotor (temporal lobe) epilepsy; management of focal or Jacksonian seizures, myoclonic jerks and akinetic attacks	Antiseizure activity per se, as do its two metabolites, phenobarbital and phenylethylmalonamide
Rufinamide (RUF) Tablet Oral solution	Adjunctive treatment in LGS (>1 ​y)	Adjunctive treatment in LGS (>1 ​y)	Voltage-gated sodium channels blocker (by stabilizing fast-inactivated state)
Stiripentol (STP) Tablet Powder for oral solution	Adjunctive in DS with CLB (>2 ​y)	Adjunctive therapy with CLB and VPA for refractory GTC in patients with severe myoclonic epilepsy in infancy	Positive allosteric modulator of GABA_A_ receptors, GABA transmission enhancer
Tiagabine hydrochloride (TGB) Tablet	Adjunctive therapy in partial seizures (>12 ​y)	Add-on therapy for partial seizures with or without secondary generalisation (>12 ​y)	GABA transporter 1 inhibitor
Topiramate (TPM) Tablet Capsule Sprinkle	Monotherapy in partial onset or primary GTC (>2 ​y) Adjunctive therapy in partial onset seizures or primary GTC seizures, seizures associated with LGS (>2 ​y)	Monotherapy in partial seizures with or without secondary generalisation and primary GTC (>6 ​y) Adjunctive therapy for partial onset Seizures with or without secondary generalization or primary GTC And for the seizures associated with LGS (>2 ​y)	Voltage-dependent sodium channels blocker, GABA transmission enhancer, AMPA/kainate receptor antagonist, carbonic anhydrase inhibitor (particularly isozymes II and IV)
Valproic acid (VPA) Tablet Oral solution Capsule Sprinkle Injection	Monotherapy and adjunctive therapy in complex partial seizures Monotherapy and adjunctive therapy in simple and complex absence seizures Adjunctive therapy in multiple seizure types that include absence seizures	Treatment of generalized epilepsy: Clonic, tonic, tonic-clonic, absence, myoclonic and atonic seizures Treatment of partial epilepsy: Partial seizures with or without secondary generalisation Treatment of specific syndromes (West, LGS)	GABA transmission enhancer, voltage-gated sodium channels blocker, T-type calcium currents inhibitor, NMDA-receptor antagonist, histone deacetylase inhibitor
Vigabatrin (VGB) Tablet Powder for oral solution	Monotherapy in infantile spasms (1 ​m–2 ​y) Adjunctive therapy in refractory complex partial seizures (>10 ​y)	Monotherapy in infantile spasms Adjunctive therapy in resistant partial epilepsy (focal onset seizures) with or without secondary generalisation where all other appropriate medicinal product combinations have proved inadequate or have not been tolerated (1 ​m–7 ​y)	GABA transaminase inhibitor
Zonisamide (ZNS) Tablet Oral solution	Adjunctive therapy in partial-onset seizures (>16 ​y)	Adjunctive therapy in partial seizures, with or without secondary generalisation (>6 ​y)	Voltage-gated sodium channels and T-type calcium channels blocker, carbonic anhydrase inhibitor

Legend: ASMs, anti-seizure medications; DRE drug-resistant epilepsy; EMA, European Medicines Agency; DS, Dravet syndrome; FDA, Food and Drug Administration; ENT-1, equilibrative nucleoside transporter 1; GPR55, (G protein-coupled receptor 55; GTC, generalized tonic-clonic; IGE, idiopathic generalized epilepsy; LGS, Lennox-Gastaut syndrome; m, month(s); NA, not available; TRPV1, transient receptor potential vanilloid 1; TSC, tuberous sclerosis complex; (XR), extended release available; y, year(s).

Sources: https://www.accessdata.fda.gov/. https://www.ema.europa.eu/.

## Mechanisms of action

### Voltage-gated sodium channels modulators

Voltage-gated sodium channels are transmembrane ion channels, highly expressed in the central nervous system (CNS) mediating Na+ ​ions influx and therefore neuronal excitability including action potentials and neurotransmitter release also tuning neuronal network functioning [20].

In mammals, sodium channels are composed of an alpha subunit associated with one or more accessory beta subunits [20]. The alpha subunit is encoded by the SCNxA genes, four of which play a crucial role in the human CNS: SCN1A, SCN2A, SCN3A, and SCN8A [21]. Their expression varies depending on the stage of development, as well as their presence may be in different neuronal populations (both excitatory and inhibitory) [22].

Phenytoin (PHT) and carbamazepine (CBZ), as well as lamotrigine (LTG), oxcarbazepine (OXC) and its metabolite, lacosamide (LCM), and eslicarbazepine acetate (ESL) (through its active metabolite S-licarbazepine) act through blockage of voltage-gated sodium channels. Other drugs, such as rufinamide (RFN), topiramate (TPM), primidone (PRM), felbamate (FBM) and zonisamide (ZNS), exert part of their activity via sodium channel blockade [23,24].

Differences in efficacy and adverse events (AEs) are not known to be due to a different binding site, which is believed to be common, but rather to a distinct affinity and binding kinetics [23].

PHT and CBZ block the fast inactivation state of voltage gated sodium channels with a 3-fold higher affinity for PHT, but 5-fold faster kinetics for CBZ [25]. OXC acts similarly to PHT and CBZ, whereas LCM and ESL are supposed to affect the slow inactivation of sodium channels [25].

Cenobamate (CNB), a new ASM approved by the FDA and EMA since respectively 2019 and 2021, enhances the inactive state of voltage-gated sodium channels by blocking the persistent sodium current. This in addition to a positive allosteric modulation of GABA_A_ receptors at a non-benzodiazepine binding site [26]. CNB is currently not approved for paediatric use [27].

Sodium channel blockers are effective for the treatment of both focal and primary generalized seizures [25]. This spectrum of action is broader for LTG, which is also efficacious for treating absences and seizures associated with Lennox-Gastaut syndrome (LGS) [25]. This is probably because, in addition to blocking the fast inactivation state of sodium channels, LTG contributes to the stabilization of presynaptic neuronal membranes and inhibits presynaptic glutamate and aspartate release [28]. In addition, LTG exerts an inhibiting action on N-type and P-type high-voltage activated calcium currents [29].

By contrast, it is well known that fast inactivation sodium channel blockers may exacerbate absences and myoclonic jerks [30]. This is less pronounced in the case of LTG, which may represent a second/third line alternative after valproic acid (VPA) and levetiracetam (LEV) for the treatment of juvenile myoclonic epilepsy (JME) [7,31]. However, the risk of myoclonic seizures exacerbation or *de novo* occurrence should always be taken into account when LTG is used in these patients [32,33].

It has also been proposed that blockade of voltage-gated sodium channels may trigger epileptic spasms in children who are at risk based on their underlying aetiology [34].

### Voltage-gated calcium channels modulators

Calcium channels are classified into high-voltage and low-voltage activated, depending on their opening threshold. Low-voltage activated channels are also known as “T-type” (“transient” or “tiny”), while high voltage-activated channels can be further classified according to their α1-subunits (CaV) subtype into L-type (CaV1.1-CaV1.4), P/Q-type (CaV2.1), N-type (CaV2.2) and R-type (CaV2.3) [35].

Activation of T-type channels in the reticular thalamic nucleus and thalamic relay neurons plays a central role in generating the pathological oscillations underlying 3 ​Hz spike-waves discharges, characteristic of childhood absence epilepsy (CAE) [36]. T-type calcium channels also appear to be involved in the intrinsic burst firing of hippocampal pyramidal neurons in temporal lobe epilepsy, as well as in nociception [37,38].

By blocking T-calcium-type currents, ethosuximide (ETH) can interrupt the oscillatory activity of thalamo-cortical circuits and thus be a very effective drug in the treatment of absences [39,40]. An inhibiting action on low-threshold T-type calcium channels is also shown by ZNS and VPA [39,41].

Originally designed as analogues of γ-aminobutyric acid (GABA), the gabapentinoid drugs gabapentin (GBP) and pregabalin (PGB) are approved for the treatment of neuropathic pain and focal seizures [42]. They can inhibit pre-synaptic high-voltage calcium currents by binding to the α2δ-1 subunit, thus reducing neurotransmitter release [43]. The higher affinity of PGB for the α2δ modulatory site explains why it is up to 6-fold more potent than GBP in animal models of epilepsy, anxiety and neuropathic pain [44]. On the other hand, GBP and PGB have the potential to induce or exacerbate myoclonus and/or absence seizures [45,46].

In addition to LTG, other broad-spectrum ASMs appear to exhibit some modulation of calcium channels. This is the case with LEV and especially VPA [23,47,48]. Not surprisingly, LTG and VPA are the drugs of choice, together with ETH, for the treatment of CAE [49]. In clinical practice, a fair proportion of patients ranging from 25% to 50% appear to respond to LEV monotherapy as well, although a seizure-worsening effect is reported in a non-negligible portion [[50], [51], [52]].

Blocking activity on some high voltage activated calcium channels is also presented by phenobarbital (PHB) and TPM [23] but also other ASMs such as CBZ and PHT [53]. Interestingly, both TPM and acetazolamide were reported to increase Calcium dependent potassium currents through an enhancement of high voltage activated L-Type calcium currents [54].

### Neurotransmitters release modulators

Synaptic vesicle glycoprotein-2 (SV2) is a family protein mediating the transport of neurotransmitters in synaptic vesicles [55,56]. This family, essential for neurotransmission, consists of 3 proteins with a high degree of homology to each other, encoded respectively on chromosome 1 (SV2A), chromosome 15 (SV2B), and chromosome 5 (SV2C) [57].

LEV and brivaracetam (BRV) primarily act by binding SV2A [58]. LEV is a broad-spectrum ASM, that is effective in both focal and primary generalized seizures [58]. Its partial effectiveness on absences has been described in *Section 3.2.2*. BRV has a 15–30 times higher affinity for SV2A, exhibiting a very similar spectrum of action [59,60]. Nevertheless, it is not currently approved for the treatment of generalized epilepsies [5].

### GABAergic transmission enhancers

GABA is the principal inhibitory neurotransmitter in the cerebral cortex which acts by binding to two types of receptors: GABA_A_, an ionotropic receptor (GABA_A_R), and GABA_B_ (GABA_B_R), a metabotropic receptor [61].

Benzodiazepines (e.g., clobazam, clonazepam, diazepam, lorazepam, midazolam) are GABA-positive allosteric modulators able to enhance the effect of synaptically released GABA, binding a specific well-characterised site at the α+-γ2– subunit interface [23]. Barbiturates (PHB) act in a very similar way and, at high doses, have also a partial agonist effect, in the absence of GABA itself. The binding site of barbiturates within the GABA_A_R remains controversial [23].

Stiripentol (STP) also operates as a GABA_A_R modulator, with a high affinity for α3- and δ-subunits [62]. Other ASMs, such as FBM and TPM, present a positive modulating action on GABA_A_R within their range of action [63].

GABA transporters (GATs) and the GABA catabolic enzyme GABA -transaminase (GABA-T) represent major inactivating systems of GABAergic transmission, removing GABA through uptake into both glia and presynaptic nerve terminals and then catabolising respectively [[64], [65], [66]].

To enhance GABA neurotransmission, vigabatrin and tiagabine were designed. They are selective irreversible inhibitors of respectively GABA-T and GAT-1 [67,68]. The former results in an overall increase in GABA levels whereas the latter prolongs the synaptic presence of GABA transiently [23].

An increase in GABA levels is also suggested for TPM and GBP, although the underlying mechanism remains unknown [69]. Lastly, it is interesting to report how other non-receptor-mediated signalling mechanisms have also been suggested for ASMs, as in the case of the VPA, which is attributed to a role in increasing GABA synthesis and reducing its degradation [70].

Also worthy of mention is ganaxolone, recently approved by FDA for the treatment of seizures associated with Cyclin-dependent Kinase-like 5 Deficiency Disorder [71].

This synthetic neuroactive steroid functions as a positive allosteric modulator for both synaptic and extrasynaptic GABA_A_ receptors, binding to a site separate from benzodiazepines or barbiturates. It has been proposed it might be able to enhance GABAergic signalling during instances when synaptic GABA_A_ receptors are internalized and benzodiazepines exhibit reduced effectiveness, as seen in refractory status epilepticus [72].

### Glutamatergic transmission inhibitors

Glutamate is the main excitatory neurotransmitter in the central nervous system (CNS), which acts by binding to 3 types of post-synaptic receptors: NMDA (N-methyl-d-aspartate), AMPA (α-amino-3-hydroxyl-5-methyl-4-isoxazole-propionate), and kainic acid [73,74]. These are 3 non-selective cation ionotropic receptors strictly linked to CNS excitability [74].

Perampanel (PER) is a non-competitive selective antagonist of AMPA receptors. Accordingly, it can reduce fast excitatory neurotransmission and thus limit the generation of seizures and the spread of seizure discharges [75,76]. Nonetheless, it is believed that PER, when administered at therapeutic doses, only blocks a minor fraction of the AMPA receptor current. This amount is adequate to slow down epileptiform discharges while preserving most of the normal synaptic transmission. This is why PER has a narrow therapeutic window, and sometimes even a slight increase in the dosage can lead to neuropsychiatric AEs [23,76].

It has been suggested that other broad-spectrum ASMs may also act partially through anti-glutamatergic activity. This is the case for FBM, which appears to inhibit NMDA receptors, and TPM, whose anti-kainate activity within the trigeminothalamic pathway may also play a role as an anti-migraine agent [77,78].

### Others

Cannabidiol (CBD) is a phytocannabinoid not producing euphoric effects and has demonstrated antiseizure activity. While the precise antiseizure mechanisms of CBD are not yet fully understood, it appears to interact with multiple signalling systems. These interactions include blocking of G protein-coupled receptor 55 (GPR55), desensitization of transient receptor potential of vanilloid type 1 (TRPV1) channels, and inhibition of adenosine reuptake. In addition, CBD has been shown to possess neuroprotective and anti-inflammatory properties [79,80].

As a result of the excellent outcomes observed in properly designed randomized controlled trials, pharmaceutically purified oral CBD is currently approved by the FDA for the treatment of seizures associated with Dravet syndrome (DS), Lennox-Gastaut syndrome (LGS), and TSC in individuals over one year of age [81]. However, the off-label use of CBD is experiencing significant growth, as it has shown efficacy rates similar to those observed in populations with LGS, DS, and TSC for other drug-resistant types of epilepsy [82,83].

The possibility that much of the antiseizure activity exerted by CBD is related to its pharmacokinetic interaction with clobazam (CLB), resulting in increased levels of both CLB and its metabolite N-desmethylclobazam, has long been debated [84]. It is currently accepted that CBD possesses independent antiseizure activity, although it may be increased when co-administered with CLB [85].

Likewise, fenfluramine (FFA) has recently been approved by the FDA for the control of drug-resistant epileptic seizures associated with DS and LGS [86]. Initially used as an appetite suppressant, this amphetamine derivative was later abandoned due to its cardiovascular side effects [87]. FFA seems to exert its antiseizure activity mainly by enhancing the GABAergic signalling through the serotoninergic pathway and by inhibiting the excitatory signalling via sigma-1 (σ1)-mediated mechanisms [88]. Although a better understanding of the pharmacological mechanisms is still needed, the effectiveness of FFA has been verified first in various animal models and subsequently in dedicated clinical trials [89,90].

Other treatment options are not strictly considered ASMs, as they have more of an anti-epileptogenic effect rather than just an anti-ictogenic action. These disease-modifying drugs include everolimus, an mTOR inhibitor, currently approved for the treatment of epileptic seizures associated with TSC [91], and cerliponase alfa for treating Ceroid Lipofuscinosis type 2 [92].

## Pharmacodynamic interactions among ASMs

Studying the pharmacodynamic relationships between various antiseizure medications can be challenging and often relies on direct clinical observation of efficacy or tolerability modifications when two drugs are combined, even in the absence of evidence of pharmacokinetic interactions [93].

It is referred to as “additivity” when the combined effects of the two drugs are equal to the sum of the individual effects, while “synergy” (or “supra-additivity”) is when the combination of the two drugs exceeds the effect of the two drugs taken individually. On the other hand, an “infra-additive” effect is observed when the combination of two drugs produces a total effect that is less than the sum of the effects produced by each drug used alone [94,95].

The association of LTG and VPA is the most documented by clinical data. Synergistic effects of this combination have been described in children with intractable typical absence seizures, in adults with a long-standing history of refractory partial seizures, and in one case of a girl with refractory myoclonic epilepsy [[95], [96], [97], [98]]. A large multicenter study in 1997, that was initially aimed to evaluate the efficacy of LTG used as an alternative monotherapy in patients treated with other ASMs (CBZ, PHB, PHT, VPA) without seizure control, also demonstrated the higher efficacy of the use of the VPA/LTG association. The initial add-on of LTG showed a higher rate of responders in patients receiving LTG together with VPA. VPA seemed to inhibit the metabolism of LTG, with a consequent increase in its plasma concentrations and a relative increase in its efficacy. However, the compensatory increase in LTG monotherapy dose following discontinuation of VPA did not match the improved efficacy achieved while patients were still taking VPA [99]. In a subsequent crossover study, 40% of patients, whose refractory complex partial seizures (focal impaired awareness seizures) did not respond to monotherapy with VPA or LTG, responded with substantial seizure reduction or seizure freedom with combination VPA/LTG. The dosages and peak serum levels of VPA and LTG used in combination were lower than when the drugs were used alone. This suggests that the major effect of this combination is not explained by the increase in serum LTG levels caused by VPA [97]. The efficacy of the combination of VPA and ETH has been demonstrated in the control of atypical absence seizures compared to monotherapy with ETH or VPA in a small case series [100] and confirmed in a retrospective study on refractory cases of absence seizures [101]. Differently from other preclinical findings, the VPA/ETH interaction can be infra-additive in decreasing the incidence of slow wave discharges in Wistar Albino Glaxo/Rat (WAG/Rij) rats [102].

Additionally, it has been proposed that LEV and LCM, probably due to their very different and non-overlapping mechanisms, may have a supra-additive effect [94].

Pre-clinical evidence also suggests the presence of a synergistic effect between VPA combined with PHT or GBP or TPM, between CBZ (or OXC) combined with GBP or TPM, between TPM combined with LTG or LEV, between LEV combined with OXC and between TGB combined with GBP [103]. Notably, the same drug combination may be synergistic or additive depending on the animal model used.

ASMs’ combination may also increase the risk of AEs, especially of neurocognitive effects. This is the case with LTG, when combined with CBZ, or with CBZ itself when combined with OXC [104,105]. The combination of two drugs with similar mechanism of action can potentially increase the risk of neurotoxicity, more than provide a benefit in seizure control [30]. As an example, in an LCM phase III study, patients taking also other sodium channel blockers experienced neurological side effects such as paraesthesia, ataxia, and coordination disorder at twice the rate of those taking ASMs with different mechanisms of action [18,106]. Additionally, the LTG/VPA combination has been associated with reports of disabling tremor and with an increased risk of skin rash, even serious (e.g., Stevens–Johnson syndrome and toxic epidermal necrolysis). However, if VPA is added to a patient taking LTG for a period long enough to become desensitized to this adverse effect, the risk of skin rash is not greatly increased [95]. Moreover, it is well-known that the co-administration of CBD and VPA leads to an increase in transaminases, which seems to be attributable to a pharmacodynamic interaction in mitochondria rather than a pharmacokinetic alteration in VPA or CBD concentrations [107].

## Drug resistance and paradoxical seizure worsening

About one-third of patients with epilepsy continue to experience seizures despite treatment with ASMs. Nevertheless, the mechanisms of DRE are currently poorly understood [108]. Different theories have been proposed to explain this phenomenon. Overexpression of efflux proteins has been postulated to affect the blood-brain barrier (BBB) in the “transporter hypothesis” or peripheral organs in the “pharmacokinetic hypothesis”. In both cases, there would be a reduction in the levels of ASMs capable of crossing the BBB. However, these two hypotheses remain controversial, and their preclinical evidence is very limited [5,103]. In the “target theory”, it has been suggested that changes in the characteristics of the drug targets associated with epilepsy could potentially lead to a decrease in drug responsiveness [108,109].

In addition, during the natural history of epilepsy, phenomena of neurodegeneration are often observed. These changes lead to the formation of new abnormal neuronal networks, increasing the risk of DRE. These are the assumptions underlying the “neural network hypothesis” [108,110].

It can also happen that sometimes ASMs are not only unable to resolve epileptic seizures, but they can also worsen them [111]. This can result from the inappropriate choice of an ASM, since CBZ, GBP, OXC, PHT, TGB, and VGB may precipitate or aggravate absence seizures, myoclonic seizures, and in some cases generalized tonic-clonic seizures. Likewise, LTG may exacerbate myoclonic seizures in patients with JME [112].

When the choice of an ASM is theoretically appropriate for the type of epilepsy syndrome but fails to control seizures, it is referred to as a “paradoxical effect” [111]. This is considered a non-specific manifestation of drug intoxication [30]. It has been suggested that this may occur more frequently with ASMs that have a limited number of mechanisms of action, such as sodium blockers or GABAergic transmission enhancers, rather than with ASMs with a broader spectrum of action, such as VPA and TPM [113,114].

## Discussion

In clinical practice, it is common to face the situation of managing polypharmacy when treating DRE. The usual practice is to start epilepsy treatment with a single, properly selected ASM with a stepwise titration, and only use combination therapy for patients who are unresponsive to two or more sequential (or alternative) monotherapies.

As shown in Table 1, regulatory agencies recommend certain considerations for selecting appropriate drugs based on the type of epilepsy (or epileptic syndrome) and patient's age. Therefore, correctly identifying the type of epilepsy (or epileptic syndrome) is essential for selecting the appropriate ASM and avoiding the negative impact on the efficacy of subsequent therapeutic attempts [115,116]. This is particularly true for patients with DRE, in whom any paroxysmal movement can sometimes be mistakenly misinterpreted as epileptic a priori.

However, when it comes to polytherapy, any combination of ASMs is theoretically possible if justified by the clinical context. Consequently, it is essential to adhere to the principles of “rational polytherapy” based on pharmacokinetic and pharmacodynamic considerations, taking into account safety and tolerability profiles. The preference for an ASM may be also guided by the presence of a specific comorbidity, which may either preclude the prescription of an ASM or suggest it, bearing in mind the effects on cognition, behaviour, mood, sedation and sleep.

Cognitive impairment is common in patients with epilepsy, due to many different contributing factors, including seizures and subclinical epileptiform activity, underlying etiology, developmental and psychological comorbidities, and the effects of ASMs (which are, among these, potentially modifiable). Although there is limited robust information specifically referring to the paediatric population and a large variability in the extent of evidence, current data suggest negative effects of PHB, PHT, TPM and ZNS on cognition, with the latter three mainly involved in language difficulties. On the other hand, notwithstanding a not clear direct role, some positive effects are associated with the use of LTG, LEV, BRV, CBD and FFA [117,118]. On the other hand, we may cite the example of the Electrical Status Epilepticus in Sleep in which early and optimal treatment with ASMs is essential in order to achieve a better cognitive outcome [119].

Furthermore, it is well known the increased risk of psychobehavioral AEs (which are associated mostly with the use of LEV, TPM and PER, but also of BRV) in patients with a history of behavioural and psychiatric comorbidities [118].

The mood stabilizing effect of VPA and LTG, as well as the anti-migraine properties of VPA and TPM can be other rational criteria for choosing them in the appropriate setting. Moreover, CBD, PER and PGB may have benefits on sleep, whereas LTG is associated with insomnia [118].

Finally, it must be considered the impact of ASMs on appetite, weight gain and growth, as a consequence. FFA, TPM, ZNS, FBM, RFN, STP, CBD, BRV and ETH are associated with reduced appetite and/or weight loss; at odds, VPA and to a lesser extent, PGB and PER, increase appetite and/or weight [120].

In general, when polytherapy is needed, it is always preferable to resort to drugs with different mechanisms of action to maximize the chances of efficacy and reduce the likelihood of poor tolerability. However, it should be considered that drugs belonging to the same class, such as sodium channel blockers, may imply some differences in channel inactivation kinetics and affinity so that even a potential combination could lead to an improvement in selected patients; although this should not be considered the first rationale approach.

The scenario is becoming particularly interesting with the advent of recent genetic advancements, which has ushered in the era of precision therapy in epilepsy. Such considerations are increasingly part of the clinical reasoning of the epileptologist, who, depending on the type of loss or gain of function mutation of a sodium channel, for example, will decide on the prescription of a sodium channel blocker.

The majority of ASMs remain anti-ictogenic. However, even this paradigm is changing with the recent introduction of drugs like everolimus that can modify the natural history of the disease.

However, despite the great expansion of our biological knowledge of epilepsy, our clinical practice is the result of empirical experience, and numerous issues remain poorly understood, such as mechanisms of drug resistance or the paradoxical worsening of seizures with an ASM. Even the most recently approved molecules, such as CBD and FFA, remain partially mysterious from the point of view of the type of mechanism of action. It is important to highlight the challenges inherent in conducting double-blind randomized placebo-controlled studies of antiseizure medications in children, especially young children. While for focal seizures the appropriateness of extrapolating data from adult trials to children from one month of age has been recently established, caution should be used in patients with mixed seizure syndromes (such as Dravet syndrome) [121].

Pharmacokinetic interactions can also influence the choice of ASMs [122]. Such extensive considerations are beyond the scope of this review, but their impact on ASMs efficacy and tolerability as well as the route administration should be always kept in mind.

A better understanding of pharmacodynamic aspects in the coming years will also lead to an improvement in the treatment of epilepsy.

We summarized the mechanisms of action of currently approved ASMs in the paediatric population, highlighting the main current evidence regarding pharmacodynamic interactions between two or more ASMs. In addition, we have outlined the current theories underlying the phenomenon of drug resistance and paradoxical seizure worsening. All these considerations are fundamental to take into account when choosing an ASM, especially in the case of polytherapy.

## Author Contributions

The authors confirm contribution to the paper as follows: study conception and design: GDO, VB, PS; literature review: GDO; draft manuscript preparation: GDO, RR, AR, ER, AV, PS. All authors reviewed the results and approved the final version of the manuscript.

## Declaration competing interest

The authors have no relevant affiliations or financial involvement with any organization or entity with a financial interest in or financial conflict with the subject matter or materials discussed in the manuscript. This includes employment, consultancies, honoraria, stock ownership or options, expert testimony, grants or patents received or pending, or royalties.

## Funding

IRCCS ‘G. Gaslini’ is a member of ERN-Epicare. This work was supported by #NEXTGENERATIONEU (NGEU) and funded by the Ministry of University and Research (MUR), National Recovery and Resilience Plan (NRRP), project MNESYS (PE0000006)—A Multiscale Integrated Approach to the Study of the Nervous System in Health and Disease (DN. 1553 October 11, 2022). IRCCS ‘G. Gaslini’ is a member of ERN-Epicare. This work was also supported by the Italian Ministry of Health, RICERCA CORRENTE 2023.
